# Creativity: a (white) matter of connectivity

**DOI:** 10.1093/cercor/bhag096

**Published:** 2026-08-20

**Authors:** Julius Voss, Karl Koschutnig, Patrick Klepits, Corinna M Perchtold-Stefan, Christian Rominger, Andreas Fink

**Affiliations:** Department of Psychology, University of Graz, Universitätsplatz 2, Graz 8010, Austria; Department of Psychology, University of Graz, Universitätsplatz 2, Graz 8010, Austria; MRI-Lab Graz, University of Graz, Kopernikusgasse 24, Graz 8010, Austria; Department of Psychology, University of Graz, Universitätsplatz 2, Graz 8010, Austria; Department of Psychology, University of Graz, Universitätsplatz 2, Graz 8010, Austria; Department of Psychology, University of Graz, Universitätsplatz 2, Graz 8010, Austria; Department of Psychology, University of Graz, Universitätsplatz 2, Graz 8010, Austria

**Keywords:** brain networks, connectometry, creativity, diffusion imaging, fractional anisotropy, quantitative anisotropy, white matter

## Abstract

In a cohort of 142 young adults, we probed how figural creative ideation maps onto white matter microstructure, aiming to challenge and refine the dominant view that creativity is driven by widely distributed brain networks. Figural creativity was assessed with the Test for Creative Thinking–Drawing Production, and its neural correlates were examined using two diffusion imaging metrics: quantitative anisotropy (QA) and fractional anisotropy (FA). Connectometry analyses uncovered robust, widespread positive associations between creative performance and white matter integrity, with the strongest effects in corticostriatal, corticopontine, and corticospinal pathways; commissural fibers such as the corpus callosum; and key association tracts including the inferior fronto-occipital fasciculus. Together, these results spotlight sensory, motor, and sensorimotor tracts as core conduits for figural creative ideation, rather than mere supporting players. QA consistently outperformed FA in detecting these creativity-related patterns, underscoring the value of deploying multiple white matter metrics in creativity research. By directly contrasting QA and FA, this study sharpens our understanding of the structural neural architecture underpinning creative processes and highlights the unique power of QA to capture microstructural features that are especially critical for figural creative performance.

## Introduction

Creative thinking is a central prerequisite for technological innovation, artistic development, and the management of complex demands (Beaty et al., 2016). Consequently, scientific interest in the processes that underlie creative ideation performance has steadily increased. Among the most intriguing research questions in this context are those that aim to uncover the neural mechanisms that underlie creativity. The present study addresses this issue by focusing on the role of white matter morphology on creative ideation, a topic that has received relatively little attention in the literature so far.

The work of Jung et al. (2013) is among the first in this field to provide a comprehensive overview of structural neuroimaging and brain lesion studies on creative cognition. This review also covers white matter and its integrity, which is central to our study. The structural connectivity of white matter determines how flexible and efficient information can be processed; it enables communication between brain regions and, thereby, may support the integration of core networks involved in creative ideation (Benedek et al., 2012; Filley and Fields, 2016). In fact, among the most important contributions of the work of Jung et al. (2013) is the notion that, in view of the increasing body of evidence, researchers should move beyond attempts to “localize” creativity in specific brain regions or single networks. Rather, the authors suggest that the creative brain is organized and supported by multiple interacting brain networks, among the most important being the default mode network (DMN) and the cognitive control network (CCN). In particular, Jung et al. (2013) were the first to propose that the DMN supports creativity through processes related to mental simulation, while interacting with control mechanisms (CCN) involved, for example, in the refinement of ideas and their selective retention. This notion has been highly influential for subsequent research and has substantially shaped the work in this field.

Subsequent neuroimaging studies, particularly those that involve resting-state brain activity, have shown that creative ideation performance is associated with the activation of multiple functional networks, in particular the DMN, the executive control network and the salience network, thus engaging a wide range of cortical and subcortical regions (Kühn et al., 2013; Beaty et al., 2014, 2016, 2019; Lloyd-Cox et al., 2022; Shofty et al., 2022). The DMN is particularly active during spontaneous and unconscious processes and plays a key role in the activation of autobiographical memories, mind-wandering, and planning future events (Otti et al., 2011; Andrews-Hanna et al., 2014). Mostly, it comprises the medial prefrontal cortex, the posterior cingulate cortex, and the lateral and medial temporal lobes (Spreng et al., 2010; Kühn et al., 2013). In contrast, deliberate control of attention is mainly subserved by the executive control network, which is located in the lateral prefrontal and anterior–inferior parietal regions (Seeley et al., 2007; Beaty et al., 2016; Dietrich, 2018; Shen et al., 2019). The interaction of these networks, particularly through the retrieval of objective-directed memories and the inhibition of dominant responses, facilitates creative ideation performance (Beaty et al., 2019). The salience network plays a mediating role, especially the right anterior insula, which identifies relevant information and regulates the switch between unconscious and conscious focus (Cocchi et al., 2013; Beaty et al., 2016).

In the context of white matter connectivity and creativity, an important question emerges: Which specific white matter tracts support the integration and interaction of neural networks that are crucial for creative ideation performance. For the DMN, key pathways include the anterior and posterior cingulum, the fornix, the corpus callosum, and the inferior and superior longitudinal fasciculi (Teipel et al., 2010; Alves et al., 2019). The executive control network is mainly supported by the superior longitudinal fasciculus, the minor forceps of the corpus callosum, and the fronto-striatal connections (Ribeiro et al., 2023). The salience network relies on connections between the anterior insula and the anterior cingulate cortex, along with the inferior fronto-occipital fasciculus (Bonnelle et al., 2012; Wang et al., 2016; Waller et al., 2017).

In line with this network-based perspective on the fiber tracts of white matter, empirical studies have provided evidence for positive associations between fractional anisotropy (FA) and creative ideation performance in bilateral prefrontal areas, corpus callosum, basal ganglia, temporoparietal junction regions, and the right inferior parietal lobule (Takeuchi et al., 2010). More recent findings also point to positive associations between FA and convergent creative thinking in the inferior fronto-occipital fasciculus and the inferior longitudinal fasciculus (Takeuchi et al., 2020). Further research has shown the involvement of subcortical pathways, including the fornix, internal and external capsules, anterior thalamic radiation, and lenticular fasciculus (Sampedro et al., 2020). Furthermore, there is evidence that structural differences in white matter may vary depending on the creativity domain. Dhakal et al. (2021) reported an increase in connectivity in the corpus callosum and cingulum in jazz improvisers, while Hong et al. (2023) found higher values of quantitative anisotropy (QA) in areas of the visual system in visual artists compared with control participants. Together, these findings underscore the importance of differentiated structural connectivity and effective functional integration for creative ideation performance in different domians.

At the same time, other studies have found that greater integrity of white matter is linked to lower performance in creative ideation. Wertz et al. (2020) demonstrated that a higher creativity index, based on divergent thinking tasks, was negatively correlated with FA in the left inferior frontal gyrus and the right uncinate fasciculus. Salvi et al. (2025) reported further negative findings on convergent creative thinking in their preprint. These findings suggest that the performance of creative ideation does not benefit exclusively from stronger structural integration, but that in certain regions reduced connectivity may promote cognitive flexibility and unconventional thinking (Jung et al., 2010; Salvi et al., 2025). The heterogeneity of the results further supports the notion that creative ideation performance is based on a complex fiber architecture in which also crossing fibers play a central role. However, classical measures such as FA are highly limited in capturing these crossing structures, as isotropic and multidirectional signal components can distort measurements (Figley et al., 2022).

In addition to established diffusion metrics such as FA, the present study, therefore, considers QA, a comparatively novel measure that has only rarely been applied in creativity research. Against this background, a direct comparison between classical and advanced diffusion measures appears to be particularly relevant. Whereas FA primarily reflects the coherence and organization of fiber tracts, QA provides a more differentiated assessment of fiber density and complex crossing structures, as the influence of isotropic signal components can be reduced in regions with fiber crossings (Basser and Pierpaoli, 1996; Yeh et al., 2010; Yeh and Tseng, 2011). Clinical studies have already confirmed this additional informational value, showing that QA captures aspects of complex fiber architecture beyond those reflected by conventional metrics such as FA, offering particular value for investigating the performance of creative ideation (Zhang et al., 2013; Yuzkan et al., 2024).

Based on this evidence, the present study analyzed the relationship between the structural integrity of white matter and creative ideation performance and examined the extent to which QA can provide insights beyond the more classical measure of FA. Special attention is paid to the tracts that support connectivity between individual brain regions within the default mode, executive control, and salience networks, while the entire brain connectome is systematically considered. We administered the widely used and well-established Test for Creative Thinking - Drawing Production (TCT-DP), which focuses strongly on aspects of quality such as content, form, composition, and elaboration (Urban, 2005). In addition, it emphasizes other factors related to creativity, including (mental) risk-taking, breaking boundaries, unconventionality, emotional expression, and humor (Urban, 2005). The primary objective of the study was to improve and expand current neuroscience research on creative ideation performance using novel measures of white matter morphology, providing deeper insight into the brain networks that underpin this domain.

## Materials and methods

### Participants

**Figure 1:**
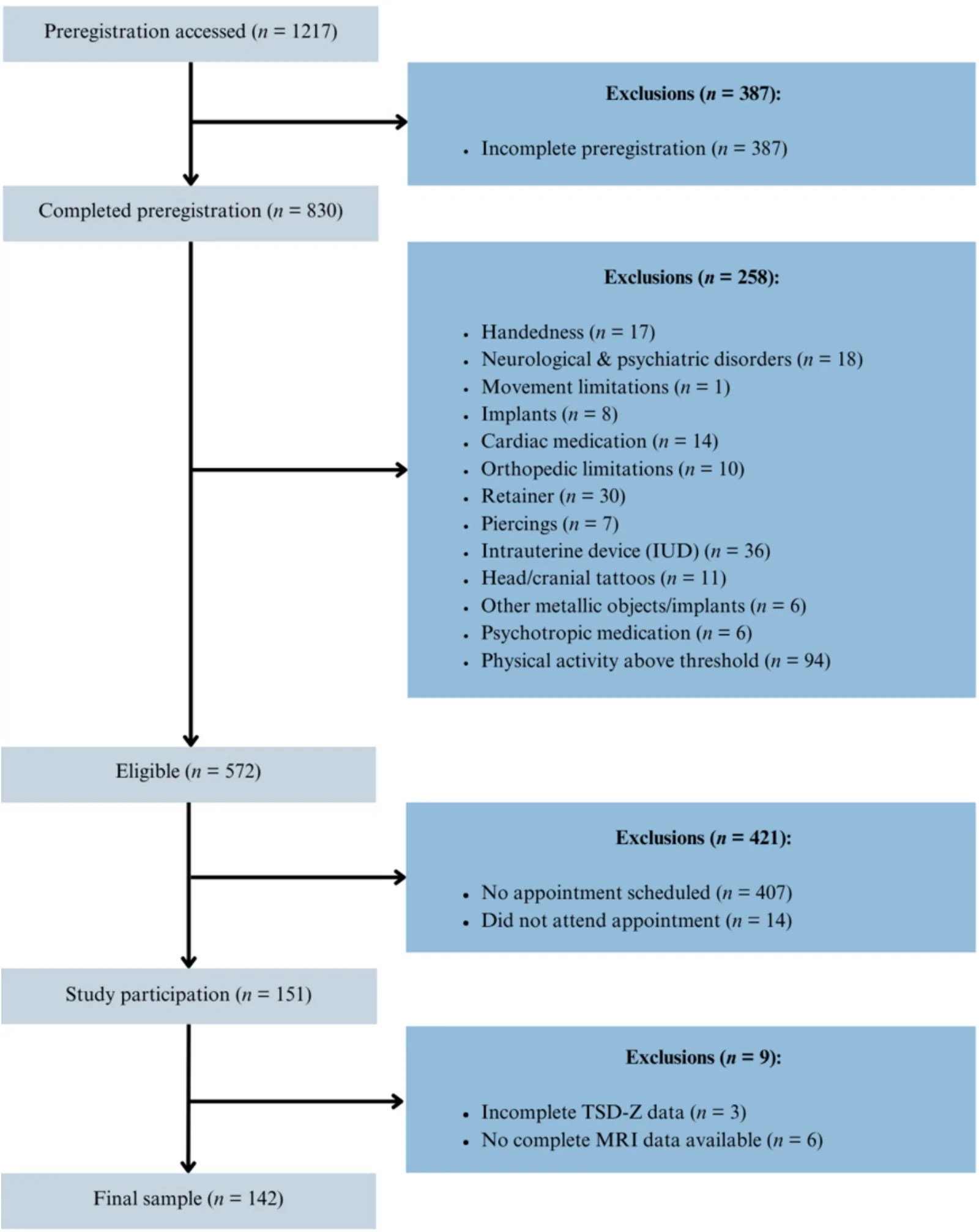
Flowchart of participant recruitment and selection. The diagram depicts the enrollment process and reasons for exclusion at each step. Of the 1,217 individuals who accessed the preregistration, 830 completed the survey. After applying predefined medical and safety exclusion criteria, 572 individuals were deemed eligible. Following scheduling and attendance of study visits, 151 participants were enrolled. The final analytic sample comprised 142 participants after excluding those with incomplete behavioral or neuroimaging data.

The final sample included 142 adults aged 18 to 38 years ($M = 24.06, {\textrm{ SD}} = 4.57$) drawn from an initial pool of 151 participants. An overview of the participant recruitment and inclusion process is presented in Fig. 1. Data from three individuals were unusable for the figural creative ideation task, and magnetic resonance data required for assessing white matter were unavailable for six participants. The resulting sample consisted of 57 men (40.1%) and 85 women (59.9%). Participants were mainly recruited through social media, printed posters, and university email lists. Most of the participants were students (62.6%). Furthermore, 26.1% indicated that they were studying and working, 9.9% were only employed, and 0.7% were in vocational training or did not report their current employment status.

As the study was integrated into a larger project on brain changes after physical activity, participants had a predominantly sedentary lifestyle and their participation in physical activity did not exceed 150 min per week. This criterion was evaluated using the *Freiburg Questionnaire of Physical Activity* (Frey et al., 1999). Only right-handed participants were eligible. Individuals with neurological or psychiatric disorders, orthopedic impairments such as knee injuries, or other limitations related to movement were excluded. Further exclusion criteria included the use of medications that affect the cardiovascular system or psychological functioning, as well as a history of heart disease. Participants with certain implants, including intrauterine devices or other medical implants, and those with metallic foreign objects such as shrapnel, nonremovable piercings, or permanent make-up were also excluded.

The study was reviewed and approved by the Ethics Committee of the University of Graz (Ethikkommission der Universität Graz; approval no. GZ. 39/55/63 ex 2022/23; approval dated 2023 February 28). Written informed consent was obtained from all participants prior to participation.

### Procedure

The entire examination within the larger project lasted several hours, and the portion relevant to the current study took about an hour. To streamline data collection, two procedural variants were used that differed in the sequence of the MRI scan and the administration of the figural creative ideation task. In one variant, magnetic resonance was performed prior to the creativity task; in the other variant, the creativity task was administered first (the two variants were approximately balanced). All participants received a verbal overview of the MRI measurement procedures and their medical eligibility was rechecked. The structural MRI itself took about 40 min.

The creativity task took place in a separate, quiet room to minimize external distractions, while the experimenter stayed in an adjacent room. The instructions were given using the standardized instruction from the manual of the *TCT-DP* (Urban and Jellen, 2010). The test sheet was positioned in the same way for all participants and the maximum time allowed for completion was 15 min. After finishing, the TCT-DP participants returned the test sheet. The actual time they had worked on the task and the title they had given their drawing were then documented.

### Instruments

#### Creative ideation performance in the figural domain

We measured figural creativity using the *TCT-DP* (Urban and Jellen, 2010). The TCT-DP is a widely used test to assess creative ideation performance in the figural domain. Participants receive a test sheet containing a square with six predrawn fragments, five of which are placed inside the square and one outside.

The evaluation of the drawings followed the test manual. Fourteen categories related to content and form were scored, including continuation of given fragments (Wf), completion of existing figures (Eg), integration of new elements (Ne), as well as figural connections (Vz) and thematic connections (Vth). Further criteria included intentional boundary-breaking (Bfa and Bfu), perspective drawings (Pe), and the inclusion of emotional or humorous aspects (Hu). Unconventionality was assessed across several dimensions, such as manipulation of the test sheet, selection of fictitious or abstract themes, use of signs and symbols, and inclusion of nonstereotypical elements. This dimension was rated across four subcategories (Uka, Ukb, Ukc, and Ukd). Processing time (Zf) was also considered an additional criterion, with shorter completion times combined with high creative ideation performance receiving bonus points. A total creativity score was calculated from the individual category scores, with a maximum attainable score of 72 points. The average creative ideation score was *M* = 25.08 (SD = 11.38), with an observed range of 6 to 55 points. The internal consistency of the individual category scores, measured using Cronbach’s alpha, was 0.74 in a sample of 145 participants available at the baseline measurement of this research project. Given the standardized instructions and clearly defined scoring criteria, high objectivity in administration and interpretation can be assumed (Urban and Jellen, 2010). In the present study, repeated administration of the test in the control group (which did not receive any intervention in the interim) enabled the computation of retest reliability across two time points: after two weeks from baseline (*n* = 41) and after four weeks from baseline (*n* = 37). The resulting correlations were *r =.49* for the two-week interval and *r = 0.61* for the four-week interval.

### Diffusion-weighted imaging data acquisition

Diffusion-weighted imaging (DWI) data were collected on a Siemens MAGNETOM Vida 3 Tesla MRI system (Siemens Healthineers, Erlangen, Germany). All scans were carried out at the MRI-Lab Graz, University of Graz, using a 64-channel head/neck coil. DWI was obtained with a monopolar single-shot echo-planar imaging sequence that was optimized to provide whole-brain coverage. Three diffusion-weighted imaging shells were acquired with b-values of 1,000, 2,000, and 3,000 s/mm$^{2}$. The number of unique diffusion directions differed between shells and increased with the b-value, from 20 directions at b = 1,000 to 30 at b = 2,000 and 64 at b = 3,000. Each shell acquisition started with a nondiffusion-weighted (b0) image. To correct for susceptibility-related distortions, an additional b0 image with reversed (anterior–posterior) phase-encoding was also collected for each shell.

All DWI-acquisitions followed the same parameters: repetition time (TR) = 3,600 ms, echo time (TE) = 109 ms, flip angle = 90$^{\circ }$. The base resolution was 106, which corresponds to a matrix size of 106 *× * 106. The slice thickness was 2.3 mm without an interslice gap. Each volume comprised 64 slices covering the whole brain. A multiband acceleration factor of 4 combined with simultaneous multi-slice acquisition was applied to reduce scan time. In the phase-encoding direction, a partial Fourier factor of 6/8 was used. Phase encoding was set from posterior-to-anterior for all scans except the additional b0 image, where the direction was reversed.

Additionally, we obtained a high-resolution structural scan for image registration. The T1-weighted image was collected with a 3D Magnetization Prepared Rapid Gradient Echo (MPRAGE) sequence using the following parameters: TR = 1,970 ms, TE = 2.06 ms, inversion time (TI) = 1,000 ms, flip angle = 8$^{\circ }$, and slice thickness = 0.9 mm. The acquisition employed a 256 *× * 256 matrix, yielding isotropic voxels of 0.9 mm. Parallel imaging was applied with GRAPPA using an acceleration factor of 2.

### MRI preprocessing

The preprocessing was performed with QSIPrep version 22.1, based on Nipype 1.9.1 (Gorgolewski et al., 2011; Cieslak et al., 2021). The T1-weighted anatomical image was aligned to the AC–PC axis and nonlinearly registered to the MNI152NLin2009cAsym template using the SyN algorithm. Skull-stripping was performed with SynthStrip. Tissue segmentation was performed using SynthSeg in combination with FreeSurfer version 7.3.1.

All diffusion volumes with b-values *100* s/mm$^{2}$ were treated as b0 images. Denoising was performed with MP-PCA. The N4 bias field correction was applied using MRtrix3 (Tustison et al., 2010; Veraart et al., 2016). Motion correction and eddy-current distortion correction were performed with FSL eddy. Susceptibility-induced distortions were corrected with TOPUP using paired b0 volumes with reversed phase-encoding directions (Andersson et al., 2003; Andersson and Sotiropoulos, 2016). Shell alignment and outlier replacement were also applied. Confounding regressors, such as framewise displacement, were computed for all datasets. Finally, DWI data were resampled to AC–PC space with an isotropic voxel size of 2 mm.

### Reconstruction and metrics

Microstructural information was extracted using restricted diffusion imaging (Yeh et al., 2017). The reconstruction of diffusion data was performed using generalized q-sampling imaging with a sampling length ratio of 1.25 (FC) (Yeh et al., 2010). In addition, tensor-based metrics were calculated from DWI data with b-values below 1750 s/mm$^{2}$. To assess white matter integrity, we computed QA and FA. QA reflects the amount of anisotropic water diffusion along the fiber orientation within a voxel and thus provides a sensitive marker of structural integrity and the density of myelinated fiber bundles (Yeh et al., 2010). FA, in contrast, reflects the degree of directional dependence of diffusion and indexes the coherence and organization of fiber tracts (Basser and Pierpaoli, 1996).

Test–retest studies in healthy subjects demonstrate that diffusion MRI-derived white matter measures show moderate to high reliability, with anisotropy metrics such as FA exhibiting the highest reproducibility (mean ICC approx. 0.70; (Duan et al., 2015)). Reliability is strongly tract-dependent: commissural fibers show the highest reproducibility, whereas projection and especially brainstem tracts exhibit lower stability. The tracts identified in our study fall within these well-characterized categories. The corpus callosum is consistently among the most reliable structures, with many tractography-derived measures demonstrating ICC *≥ * 0.70 and coefficients of variation *≤ * 10. Similarly, long association tracts such as the inferior fronto-occipital fasciculus and inferior longitudinal fasciculus show good to excellent test–retest reliability (ICC approx. 0.62 to 0.95) in HARDI-based tractography studies (Boukadi et al., 2019). In contrast, projection pathways such as thalamic radiations and corticopontine tracts show more moderate reliability, while brainstem tracts (eg medial lemniscus) are known to exhibit lower reproducibility due to their small size and susceptibility to imaging artifacts. Although fewer studies have directly evaluated QA, anisotropy-based measures are generally the most reproducible class of diffusion metrics. Given that QA is derived from the spin distribution function and is less sensitive to crossing fibers than tensor-based indices, it is expected to exhibit comparable or potentially improved reliability, particularly in anatomically complex regions.

### Statistical analysis

To examine the relationship between creative ideation performance and white matter integrity, connectometry analysis was performed following the procedure of Yeh et al. (2021) using DSI Studio (DSI Studio version: Hou, 2025 September 4) (Yeh, 2025). Nonparametric Spearman correlations were calculated between the total score of TCT-DP and the extracted diffusion metrics (QA and FA). The quality of normalization (fitness $R^{2}$), along with sex and age, was included as a covariate to account for potential noise effects. The thresholds for the correlation coefficients were set at *ρ * = 0.25 (corresponding to a t value of 3.06 for *n* = 142) with a minimum tract length of 25 voxels. The chosen thresholds should ensure sufficient data quality and specificity by capturing anatomically plausible bundles with meaningful small to moderate associations with creative ideation performance. To reduce topologically implausible connections, topology-informed pruning with 16 iterations was used (Yeh et al., 2019). The False discovery rate correction with *q* < 0.05 was applied to control for multiple comparisons. The null distribution of tract lengths was determined by 4,000 permutations (Yeh et al., 2016). The analysis parameters followed the methodological standards of a recent study on white matter (Kirby et al., 2024). To capture a comprehensive range of effect size and length threshold combinations, we calculated the results for QA and FA using effect sizes from 0.2 to 0.3 (step size 0.1) and length thresholds from 25 to 60 (step size 5). This provides a transparent overview of results across a reasonable spectrum of parameter combinations (see Interactive Viewer – supplement).

In addition, fiber tracts were categorized in DSI Studio into projection, association, commissural, and cerebellar fibers and included in subsequent analyses. This classification follows established neuroanatomical nomenclature, with association fibers connecting cortical regions within a hemisphere, commissural fibers linking regions across hemispheres, projection fibers connecting cortical and subcortical structures, and cerebellar fibers located within the cerebellum.

## Results

### Quantitative anisotropy

**Figure 2:**
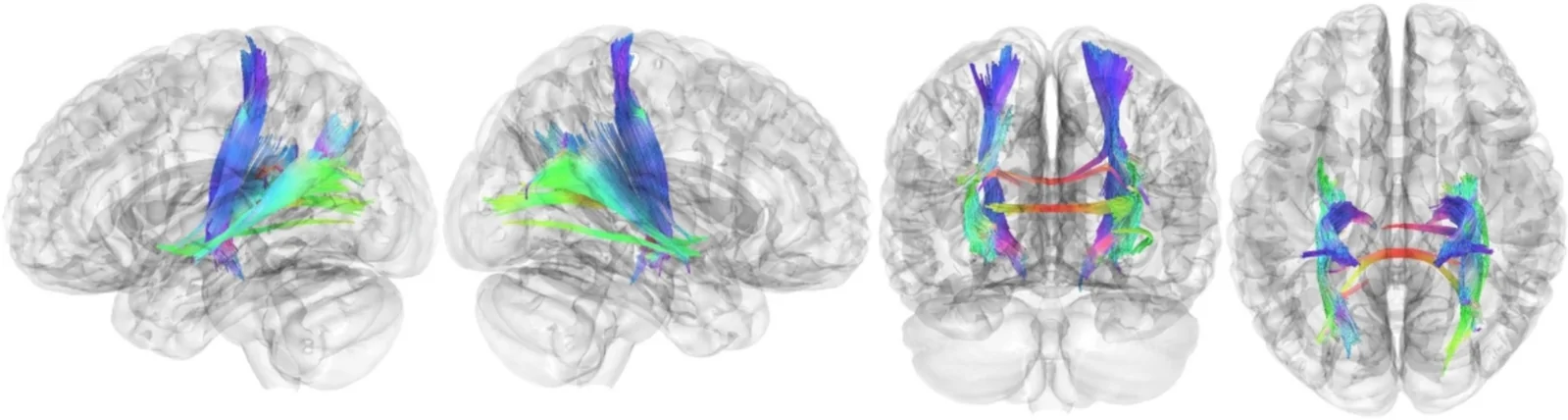
White matter tracts showing significant positive correlations (*ρ * = 0.25 and tract length > 30 voxels) with creative ideation performance for QA. Tractography is visualized using directional color-coding.

At *ρ * = 0.25 and a minimum tract length of 30 voxels, several fiber tracts exhibited significant positive correlations with creative ideation performance. No negative associations were found with these thresholds (see the interactive viewer in the supplemental material). The identified tracts mainly involved projection fibers, especially in the bilateral corticopontine, corticostriatal, and corticospinal tracts, as well as the left medial lemniscus, as summarized in Table 1 and Fig. 2. Additionally, the analysis revealed positive associations between creative ideation performance and association fibers, including bilateral inferior fronto-occipital fasciculi, along with commissural fibers such as the forceps major of the corpus callosum.

**Table 1:** Tract counts.

System	Tract	Region	Count
Projection brainstem	Corticopontine tract	Occipital (L)	272
Projection basal ganglia	Corticostriatal tract	Post. (L)	229
Projection brainstem	Corticospinal tract	R	223
Projection brainstem	Medial lemniscus (L)		201
Projection brainstem	Corticopontine tract	Parietal (R)	200
Projection basal ganglia	Corticostriatal tract	Post. (R)	177
Projection brainstem	Corticopontine tract	Parietal (L)	159
Projection brainstem	Corticopontine tract	Occipital (R)	144
Association	Inferior fronto-occipital fasciculus (L)		140
Association	Inferior fronto-occipital fasciculus (R)		139
Projection basal ganglia	Thalamic radiation	Post. (L)	120
Projection basal ganglia	Corticostriatal tract	Sup. (R)	97
Association	Inferior longitudinal fasciculus (R)		85
Commissure	Corpus callosum	Forceps major	74
Projection brainstem	Corticospinal tract	L	73
Projection brainstem	Medial lemniscus	Medial lemniscus (R)	60
Association	Extreme capsule (L)		43
Commissure	Corpus callosum	Body	28
Projection basal ganglia	Corticostriatal tract	Sup. (L)	27
Commissure	Corpus callosum	Tapetum	18

L, left; R, right; Post., posterior; Sup., superior.

### Fractional anisotropy

At *ρ * = 0.25 and a minimum tract length of 30 voxels, no significant associations with creative ideation performance were detected. The interactive connectometry viewer (see supplemental material) indicates some weak positive associations (primarily involving the corticopontine and corticostriatal tracts), all of which were below the chosen threshold values.

## Discussion

The present study investigated the association between indices of white matter microstructure and creative ideation performance, with a particular focus on the additional explanatory value of QA compared with the classical diffusion metric of FA. The results consistently indicate that the microstructure of the white matter, measured by QA, is positively associated with creative ideation performance in the figural domain.

Specifically, QA was positively associated with a network of projection fibers involving the corticostriatal, corticopontine, and corticospinal tracts. These descending pathways connect the cortex to the basal ganglia, the pons, and the spinal cord, respectively, and are known primarily for their roles in motor behavior and motor control (Lemon, 2008). Despite this, neuroscience studies in human participants reveal a broader role for these pathways, collectively supporting various sensory, cognitive, affective, and motor processes (Seger, 2013; Haber, 2016). For instance, corticostriatal connections are believed to support appropriate goal-directed behaviors (Haber, 2016), mood functions (Admon and Pizzagalli, 2015), visuomotor learning (Park et al., 2024), and creative imagery (Rahmani et al., 2020). Similarly, corticopontine connections have been implicated in mental flexibility (Anziano et al., 2023). In addition, the corticospinal tract and medial lemniscus are closely related to sensorimotor processes (Yang et al., 2009; Welniarz et al., 2016).

**Figure 3:**
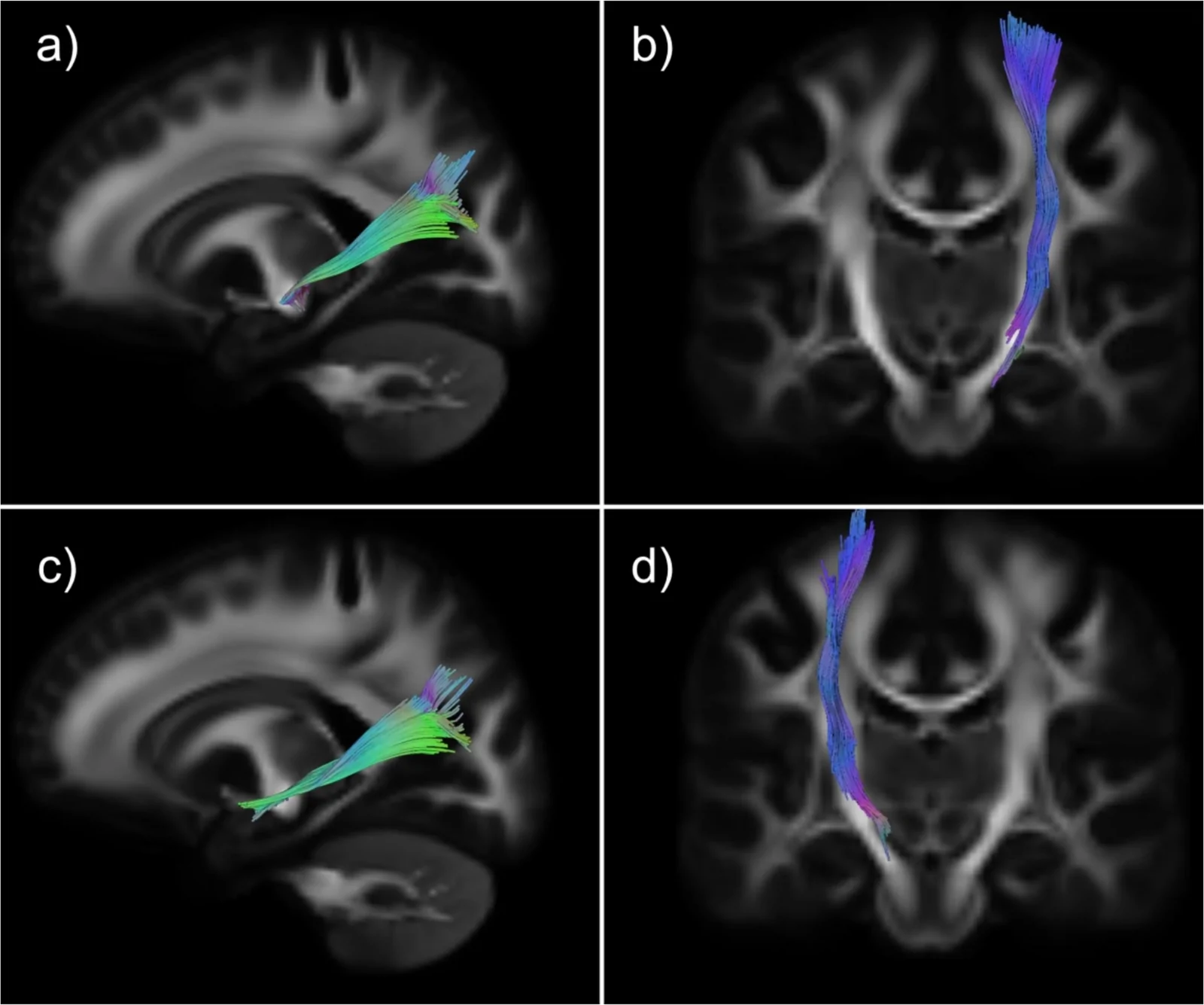
Anatomical reconstruction of major white matter projection pathways. Subfigures illustrate the trajectory and connectivity of four identified tracts: (a) left occipital corticopontine tract, (b) right corticospinal tract, (c) left posterior corticostriatal tract, and (d) left medial lemniscus. Numerical values represent the streamline count for each respective tract: (a) 272, (b) 223, (c) 229, and (d) 201. L, left; R, right; Post., posterior.

Critically, the literature suggests clear functional specializations of these pathways depending on the cortical regions from which they receive input (Karbasforoushan et al., 2022). As indicated by the results of this study (see Figs 2 and 3), both the corticostriatal and corticopontine pathways identified here arise from posterior/occipital cortical areas, suggesting that sensory or visual input plays a crucial role in this form of creativity. These findings not only align with studies that attribute a role to motor simulations and sensorimotor networks in the generation of creative ideas (Dietrich, 2018; Rominger et al., 2018; Sampedro et al., 2020), but also suggest that sensory, motor, and sensorimotor networks may play an especially significant role when creative ideation tasks require both cognitive and motor processes, as is the case for figural creativity and drawing.

Although the findings of this study revealed a network that appears to be highly specific to creativity in the figural domain, they also complement broader findings in the general domain of creativity. In particular, the observed associations of creative ideation performance with commissural fibers, such as the corpus callosum, and association pathways involving the right inferior longitudinal fasciculus and the bilateral fronto-occipital fasciculi clearly indicate that creativity is supported by connectivity across different networks. These pathways are particularly involved in the default mode, executive control, and salience networks, which are critical components of this architecture (Teipel et al., 2010; Waller et al., 2017; Alves et al., 2019). Collectively, these findings support current models that emphasize the flexible integration of different brain networks as a central prerequisite for creative processes (Kühn et al., 2013; Beaty et al., 2014; Lloyd-Cox et al., 2022; Shofty et al., 2022).

At the chosen thresholds of *ρ * = 0.25, the analysis of FA did not produce significant findings. Some positive associations between FA and figural creativity were identified at lower threshold values, and these largely overlapped with the QA findings (see the interactive viewer in the supplemental material). However, these effects were smaller in magnitude and did not exceed the threshold values of *ρ * = 0.20 and a length threshold of 25 voxels. In particular, no negative associations were observed between FA and figural creativity, reflecting the results with the QA index.

Taken together, the present results confirm the hypothesis of a positive association between creative ideation performance and the structural integrity of white matter, supporting the assumption that large-scale distributed networks form the foundation of creative cognition. Although the effect sizes were small to moderate, they nonetheless highlighted the relevance of highly specific sensory, motor, and sensorimotor networks, along with more general association and commissural fibers, for creative ideation in the figural domain. Regardless of the diffusion metric used, consistently positive associations emerged between white matter morphology and creative ideation performance, underscoring the overall importance of greater network integrity for creativity. These findings stand in contrast to earlier reports of negative associations with FA (Jung et al., 2010; Wertz et al., 2020), which were predominantly associated with convergent creativity and tasks focused on verbal creative ideation (Salvi et al., 2025).

A central contribution of this study is the direct comparison of QA and FA. Although both measures showed associations with creative ideation performance, QA demonstrated a higher sensitivity, especially in terms of the observed effect sizes. This finding is consistent with previous research showing that QA provides additional information in regions with complex (ie crossing) fiber architecture (Yeh et al., 2010; Zhang et al., 2013; Figley et al., 2022; Yuzkan et al., 2024). Whereas FA primarily reflects the coherence and organization of fiber tracts, QA captures the density of anisotropic diffusion along specific fiber bundles (Basser and Pierpaoli, 1996; Yeh et al., 2010). Consequently, both measures reflect different but complementary aspects of the microstructure of white matter. This complementarity also became evident in the present findings, as QA revealed broader and stronger associations with creative ideation performance, while FA identified—though not exceeding the chosen threshold—some partially distinct tracts that point to additional functional contributions. Therefore, the present findings suggest that creativity is not determined solely by the coherence of individual fiber tracts. Rather, microstructural properties such as the density and integrity of specific fiber pathways, which are captured more precisely by QA, appear to play an important role. Denser and more efficiently organized connections may functionally support more flexible interactions within large-scale networks and thereby facilitate creative ideation. Future research should systematically integrate both measures, with particular attention to QA, to unravel the contributions of fiber density and coherence, and to better understand how structural connectivity supports the dynamic interaction of large-scale brain networks underlying creative ideation.

### Limitations

Despite these findings, several limitations must be noted. The sample consisted primarily of young adults with a high level of education, which restricts the generalizability of the results. In addition, we assessed creative ideation performance exclusively in the figural domain. Although this measure captures both quantitative and qualitative dimensions, future studies would benefit from combining different instruments that assess divergent and convergent thinking, as well as various forms of creative expression, such as writing and improvization. Furthermore, the cross-sectional design precludes causal inferences. Longitudinal and multimodal approaches may be crucial to more clearly determining both the stability and functional relevance of the observed associations.

While this study can be considered a first demonstration of how figural creativity is generally related to this novel measure of white matter morphology, future research is needed to examine whether women and men show similar or distinct relationships between QA and creativity. Preliminary analyses conducted with the present sample indicate that women and men may possibly exhibit different patterns of associations; however, with 57 men and 85 women, the gender distribution in this study is imbalanced, and this imbalance in sample size may also affect the QA findings, complicating the investigation of potential gender differences. We very much hope to see more gender-balanced samples in future studies that will explore gender effects in greater detail.

Another critical issue is that the specificity of the QA–creativity relationship needs to be demonstrated in future research. For example, recent meta-analyses show that divergent thinking is significantly correlated with intelligence (Gerwig et al., 2021). At the same time, DTI measures have been found to be related to intelligence (Colom et al., 2010). In the present study, QA was positively associated with a network of projection fibers involving the corticostriatal, corticopontine, and corticospinal tracts. As these tracts are known primarily for their roles in motor behavior and motor control, these processes might be particularly relevant for figural creativity. However, this study also revealed tracts that support more general cognitive functions. Therefore, future research should assess the unique contribution of creativity to DTI measures beyond IQ.

## Conclusion

The present study adds to the literature by supporting the involvement of large-scale brain networks in creative ideation and by identifying associations between figural creative ideation and white matter tracts in sensory, motor, and sensorimotor systems. By comparing FA with QA, the findings underscore the added value of QA as a more sensitive and complementary metric to investigate the microstructural white matter foundations of creativity. Overall, the results are consistent with the notion that the performance of creative ideation is not driven by single dominant connections, but rather emerges from the dynamic interplay of numerous partially overlapping network resources.

These insights support existing research by showing that creativity is based on distributed and flexible structural connectivity, while also offering a potential explanation for previous inconsistencies in studies based exclusively on FA. These findings also highlight that integrating multiple diffusion metrics in future research (Koschutnig et al., 2024), along with multimodal, longitudinal, and domain-diverse assessments, can provide a more comprehensive understanding of the structural underpinnings of creative ideation.

## Supplementary Material

Web_Material_bhag096

## Data Availability

All data underlying the conclusions of this study are accessible on the Austrian Neuro Cloud https://doi.org/10.60817/sama-va10.
