# Supplementary material for: Creativity: a (white) matter of connectivity
Source: Cereb Cortex. 2026 Aug 20;36(8):bhag096. doi: 10.1093/cercor/bhag096 (PMC13490752; doi:10.1093/cercor/bhag096)
Supplement: Supplementary file 1 [file web_material_bhag096.zip › Interactive Viewer.html]

Connectometry Interactive Viewer


## Connectometry Interactive Viewer

MRI-Lab Graz  
Karl Koschutnig  
karl.koschutnig@uni-graz

**Color legend (DTI/DWI direction):**  
Red: Left–Right (transverse)
  
Green: Anterior–Posterior (front–back)
  
Blue: Superior–Inferior (up–down)
  
Yellow (red+green): anterolateral
  
Cyan (green+blue): superolateral
  
Magenta (red+blue): oblique

Modality

Type

Increase
Decrease
Effect size


Threshold
